# Editorial: Parental mentalization: New frontiers

**DOI:** 10.3389/fpsyg.2023.1154250

**Published:** 2023-03-28

**Authors:** Mirjam Kalland, Helena Rutherford, Marjaterttu Pajulo

**Affiliations:** ^1^Faculty of Educational Sciences, Department of Educational Sciences, University of Helsinki, Helsinki, Finland; ^2^Yale School of Medicine, Child Study Center, Yale University, New Haven, CT, United States; ^3^Department of Clinical Medicine, University of Turku, Turku, Finland

**Keywords:** parental mentalization, reflective functioning, child development, maltreatment, embodied mentalization, treatment outcome

Central to navigating social interactions is the ability to interpret and predict behaviors through understanding the underlying thoughts, feelings, and emotions of the self and others—referred to as mentalization (Fonagy et al., 1991). Early attachment relationships are thought to lay the foundation for mentalization (Fonagy et al., 2002), and disruptions in mentalization have been associated with psychopathology (Fonagy and Luyten, 2009). Importantly, mentalization may be dynamic and relationally specific. Parental mentalization is thought to play a critical role in child development, scaffolding the child's own ability to regulate their emotions to support the later emergence of their own ability to mentalize (Slade, 2005). Infants are limited in their non-verbal communicative bids. Consequently, parents may need to flexibly adapt their mentalizing capacity to understand infant affective cues to sensitively respond to their child, supporting the emerging security of the early attachment relationship. Importantly, intervention research has shown that mentalization-based work with at-risk parents have a greater potential to enhance parent-child interactions and greater attachment security in comparison with psycho-educational approaches (Suchman et al., 2017).

Despite parental mentalization being a core theme of clinical work, it remains a relatively new construct in the scientific literature, with many exciting avenues for research. Due to demanding and time-consuming methodology in the measurement of parental mentalization, sample sizes in empirical research have been limited. Therefore, there is great need to develop more feasible, but accurate, measures of parental mentalization that can be scaled to larger samples. There is also significant heterogeneity in measures of parental mentalization that have primarily focused on mothers, and not fathers, limiting the generalization of this work. With respect to intervention studies, more use of controlled research designs, especially randomized clinical trials designs is needed alongside longitudinal approaches. It is also important to broaden the focus of child developmental outcomes, including empathy and the child's own capacity to mentalize. Our goal in this Research Topic was to showcase the most essential advances and research projects currently going on in the field of parental mentalization.

## Parental mentalizing: Research presented in this Research Topic

The Research Topic consists of seven papers, with 36 authors included: four original research articles, one systematic review, and two brief research reports. One of the emergent themes of the papers presented are the varying approaches to measuring parental mentalization, which has often been operationalized as parental reflective functioning: an overt manifestation of the mental processes that underpin the capacity to mentalize at an individual level as a parent as well as when interacting with the child (Nijssens et al., 2020). Historically, the assessment of parental mentalization has been captured by interview-based approaches that are then scored by reliable coders for the level of reflective functioning that parents express through the course of the interview. The two main interview approaches have been the Adult Attachment Interview (AAI, George et al., 1984) and the Parent Development Interview (PDI, Slade et al., 2004). Both the AAI and PDI have been designed to assess parental capacity of mentalizing *via* a transcribed, semi-structured interview. However, while PDI questions focus on current parenting of the child, the AAI focuses on the childhood experiences. Traditionally, researchers implement either the AAI or the PDI; however, in the current issue, Flykt et al., employed both measures to examine parental reflective functioning. Their central finding was that reflective functioning as measured by the AAI and reflective functioning as measured by the PDI seem to be partly distinct constructs of a broader parental mentalizing capacity, and therefore could be uniquely targeted in interventions. These findings are valuable, as research has not yet fully differentiated the interconnections and different outcomes of reflective functioning in the context of these two interview approaches.

Two further papers in the Research Topic separately examined the AAI and PDI in clinical samples. In the paper presented by Rosso, parental RF was assessed using the AAI among parents who were known to have maltreated their children. These parents were found to have severe impairments in their ability to mentalize upon their own childhood experiences. In most cases, their reflective capacity was not only absent, but they systematically resisted taking a reflective stance. Suboptimal mentalizing capacity assessed by the PDI was also found among mothers using substances who had a childhood history of their own mother's substance abuse and mental illness (Lowell et al.). Somewhat surprisingly, mothers with childhood experiences of their own mother's mental illness *and* substance use had higher levels of PRF in comparison to mothers with a background of maternal substance use alone.

Although largely considered gold-started assessments of parental mentalization, interviews designed to assess PRF are time consuming to administer and interpret, and therefore attempts have been made to develop “off-line” self-report instruments, such as the Parental Reflective Functioning Questionnaire (PRFQ, Luyten et al., 2017), to make it possible to evaluate parental RF in large samples. However, self-report questionnaires are limited in their ability to catch the rich and multilevel concept of PRF and may be limited by responder bias.

Questionnaire measures also need to be carefully evaluated in different cultural contexts. With this in mind, work by Ye et al. in this issue successfully implemented the PRFQ in Chinese parents. The confirmatory factor analysis indicated that the Chinese version of the PRFQ with 12 items is psychometrically sound and can be applied to populations in China. Furthermore, this issue also includes the application the PRFQ self-report questionnaire in assessing the early unfolding of maternal mentalization for the first time in a large Finnish cohort (Lindblom et al.). Importantly, it was found that the “high” maternal mentalization profile in early parenthood was associated with high child socio-emotional competence at the age of two years, including the child's capacity for empathy.

A more recent advance in the measurement of parental mentalization that examines non-verbal behavior, instead of relying on parents' verbal expressions, is presented in this issue. Shai evidenced the positive impact of parental embodied mentalization on children's cognitive and language development in a longitudinal study. Such findings are important as they indicate the predictive value of both verbal and non-verbal approaches in the assessment parental mentalization.

Finally, in their systematic review included in this issue, Stuhrmann et al. explored how PRF is associated with parenting behaviors incorporating only studies using the PDI. Based on their review they concluded that most of the associations indicated a positive effect of PRF on parenting quality further emphasizing parental mentalization as a critical construct for parent and child development. However, the complex interaction between PRF and contextual factors emphasizes the need for differentiation of PRF dimensions.

## Perspectives on future research

In conclusion, this Research Topic provides more insight to the ongoing research activity in the field of parental mentalization, highlighting its importance, as well as its methodological development. In the future it will be important to better understand and identify the different forms and aspects of parental mentalizing, as well as different and overlapping dimensions between different measurement approaches. This will be especially valuable for designing and interpreting the results of intervention studies that are designed to enhance parental mentalization.

## Author contributions

MK and HR wrote the first draft of the manuscript. MP commented and added to sections of the manuscript. All authors contributed to manuscript revision, read, and approved the submitted version.
